# The social odor scale: Development and initial validation of a new scale for the assessment of social odor awareness

**DOI:** 10.1371/journal.pone.0260587

**Published:** 2021-12-14

**Authors:** Elisa Dal Bò, Claudio Gentili, Andrea Spoto, Giovanni Bruno, Andrea Castellani, Carmen Tripodi, Florian Ph. S. Fischmeister, Cinzia Cecchetto

**Affiliations:** 1 Padova Neuroscience Center (PNC), University of Padua, Padua, Italy; 2 Department of General Psychology, University of Padua, Padua, Italy; 3 Institute of Psychology, University of Graz, Graz, Austria; 4 Department of Biomedical Imaging and Image-Guided Therapy, Medical University of Vienna, Vienna, Austria; University of Bologna, ITALY

## Abstract

The degree of attention individuals pay to olfactory cues (called odor awareness) influences the role of odors in everyday life. Particularly, odors produced by the human body (i.e., social odors) are able to carry a wide variety of information and to elicit a broad spectrum of emotional reactions, making them essential in interpersonal relationships. Hence, despite the assessment of awareness toward social odors is crucial, a proper tool is still lacking. Here, we designed and initially validated the Social Odor Scale (SOS), a 12-item scale designed to measure the individual differences in awareness towards different social odors. In Study 1, an exploratory factor analysis (EFA; KMO test: MSA = 0.78; Bartlett’s test: χ^2^(78) = 631.34, p < 0.001; Chi-squared test: χ^2^(42) = 71.84, p = 0.003) suggests that the three factors structure was the model that best fit with the Italian version of the scale. The confirmatory factor analysis (CFA) supports a second-order model with one higher-order factor representing social odor awareness in general and three lower-order factors representing familiar, romantic partner, and stranger social odors. The final version of the scale presented a good fit (RMSEA = 0.012, SRMR = 0.069, CFI = 0.998, TLI = 0.997). In Study 2, CFA was performed in the German version of the scale confirming the validity of scale structure. Study 3 and 4 revealed that SOS total score and its subscales were positively correlated with other validated olfactory scales, but not with olfactory abilities. Moreover, SOS was found to be related to the gender of the participants: women reported to be more aware to social odors and, specifically, to familiar social odors than men. Overall, the results indicated that SOS is a valid and reliable instrument to assess awareness toward social odors in everyday life.

## Introduction

The prominent role of odors in people’s life is well known [1], however not all humans pay attention to odors in the environment in the same way [2]. There are individuals that spontaneously notice the food aroma or the fragrances of flowers, whereas others become aware only after drawing their attention to them. This meta-cognitive ability has been called “odor awareness”, reflecting the degree to which individuals catch olfactory cues and let them guide and affect their attitudes and actions [2]. Odor awareness was found to be related to both the self-rated sense of smell (the better participants ranked their ability to smell, the better their olfactory awareness) and to olfactory abilities (people who reported to be less aware of odors had a significantly poorer olfactory performance than individuals claiming a higher odor awareness) [2].

Odor awareness is relevant not only for the examples described before but also for odors, especially body odors, conveying socially relevant messages. Indeed, as in other species [3], increasing evidence suggests that the chemicals produced by the human body are essential in interpersonal relationships [4, 5]. Essentially, each individual presents a typical body odor that, as for physical appearance, reflects personal stable characteristics or transient events (e.g., personality, sex, age, health, and even transient emotional states; [6, 7]). These odors are perceived by other people and affect their reaction: they become social odors [8, 9]. For example, the “smell of sickness” (i.e., sweat collected during a sickness condition) can influence social desirability [10] or the “smell of fear” (i.e., sweat collected during a fearful condition) can affect behavior [6, 11].

The processing of social odors mostly occurs without the allocation of attention, modulating individuals’ behaviors unconsciously [12–15]. However, it has been demonstrated that humans are able to partially distinguish familiar to unfamiliar social odors consciously [16–19]. The degree to which odors are important in social contexts can change across individuals. For instance, Sorokowska and colleagues [20], analysing the odor awareness toward interpersonal situations of individuals from 44 countries, demonstrated that social odors are more strongly related to individuals’ characteristics, i.e., gender, age, and education, than cultural or country factors.

Despite the growing body of literature documenting the importance of human social odors on everyday social interactions [4], a proper and specific tool that measures individual differences in the awareness toward social odors is still missing. Indeed, existing questionnaires, which for example assessed the awareness toward odors (the Odor Awareness Scale, OAS, [2]), or the importance that people give to the sense of smell (the Individual Significance of Olfaction scale, ISO, [21]), or the attitudes toward odors in everyday life (the Odours in Everyday Life Questionnaire, OELQ, [22]), include both items that assess the perception of common odors and the perception of bodily odors in interpersonal situations. Recently, the Body Odor Disgust Scale (BODS, [23]) was developed. However, as specified in its name, it refers exclusively to the emotion of disgust. Yet, previous literature has shown that social odors are able to carry a wide variety of information and to elicit a broad spectrum of emotional reactions (e.g., attractiveness, fear, safety; [6, 24, 25]).

Given the lack of a tool that specifically assesses social odor awareness, we present here a series of studies designed to develop and validate a new scale called Social Odor Scale (SOS). By reviewing the previous literature on social odors and the existing olfactory scales [2, 21–23] we could identify four types of social odors that occur in individuals’ everyday life. First, romantic partner odor: social odors play an important role in mate selection by affecting desirability [10, 26–28] with this process being only partially subconscious, as both men and women seem aware of using odors in their mate choice [29]. Second, odors of familiars or significant others: these social odors provide a feeling of security and familiarity and help the relationship maintenance [30–34]. Familiar social odors include odors from significant others, family members, and friends [16, 18, 35–37]. Third, stranger’s body odors: these odors are processed as a dangerous and threatening stimulus and they are rated as more unpleasant and intense than a friend’s body odor [16, 19, 38]; and finally, fourth, own social odor: people can discriminate their own body odor from the odors of others and thus constantly smell themself as a reassuring behavior to obtain information on individuals with whom there has been contact which in turn provides information about the self [39, 40]. The SOS has been specifically developed to measure the level of attention participants pay to the four types of social odors (own, familiar, romantic partner, and stranger) and thus we expected a four-factor questionnaire.

In Study 1 an Exploratory Factor Analysis (EFA) and a Confirmatory Factor Analysis (CFA) were conducted to assess and confirm the factor structure of the Italian version of the scale. In Study 2 the CFA was replicated on the German version of the SOS. In Study 3 we measured the association between the SOS and the actual olfactory abilities measured with the Sniffin’ Sticks test [41]. In Study 4 we investigated the relationship between the SOS and demographic variables and self-reported olfactory abilities.

## Study 1: Exploratory factor analysis and confirmatory factor analysis on the Italian version

Study 1 was designed to investigate the factor structure of the Italian version of the scale through the exploratory factor analysis (EFA) and to test the validity of the proposed model through the confirmatory factor analyses (CFA).

### Material and methods

#### Item construction

Four types of social odors were identified by reviewing the previous literature and the existing olfactory scales [2, 21–23]: individual body odor, familiar and unfamiliar body odor, romantic partner body odor, and body odor of strangers. Items were designed to assess the awareness of body odors (the person’s tendency to pay attention to odors), behavior (avoidance or approach), and emotions (e.g., annoyed, attracted, aroused) related to body odors. Each item was formulated as a personal statement. Initially, a pool of 30 items was created: 20 items were formulated by revising and rephrasing items from other chemosensory scales: the OELQ [22], the OAS [2], the BODS [23], and the ISO [21]. Other 10 specific scenarios, in which the individual could be affected by body odors, were set. Finally, the authors reviewed this pool to have 6 items for each type of body odors. The criteria were appropriateness (meaning that each item correctly described a scenario in which the specific body odor was involved), comprehensibility (meaning that each item was clear and easily understandable by readers), and redundancy (meaning that each item was not too similar to the other items). These 24 items were then presented to three experts, who were not part of the project, who agreed that each item was appropriate, clear, and not redundant. Their judgment was only qualitative.

For each item, participants had to rate to what extent they agree or disagree with each of the statements presented. The scoring was obtained using a 5-point Likert scale (0 = I totally disagree, 1 = I mostly disagree, 2 = I neither agree nor disagree, 3 = I mostly agree, 4 = I totally agree).

#### Participants

The present study was part of a broader project that aims to explore if the awareness of body odor is related to the olfactory ability, awareness, and imagery, as well as to several psychological characteristics. The survey was presented online using the Qualtrics survey platform (Qualtrics XM Platform). Participants between 18 and 45 years old were recruited via social media, word of mouth, and during classes at the University of Padua and the University of Pisa. A total of 533 Italian-speaking participants took part in this study. Participants were excluded if they were diagnosed with COVID-19 [42] and if they were pregnant or breastfeeding. Moreover, we excluded participants who did not answer correctly to the control item that was included to control the tendency of some participants to answer items without reading the content [43, 44]. Therefore, the final sample was composed of 343 participants (259 women, 78 men, 6 did not specify their gender), between 18–45 years (mean = 24.8; SD = 5.5).

The present study was conducted with the adequate understanding and digital consent of the participants in accordance with the Declaration of Helsinki and was approved by the local Ethics Committee, University of Padua (prot. no. 3667).

#### Statistical analyses

Data were cleaned using the software R [45] and analyzed with JASP [46]. The sample was randomly split into two datasets of approximately equal size and with equal number of men and women: the Exploratory Factor Analysis (EFA, N = 171) was performed on one half, the Confirmatory Factor Analysis (CFA, N = 172) was performed on the other half. CFA was performed with the software R using the packages “lavaan” [47].

### Results and discussion

The initial Kaiser–Meyer–Olkin (KMO) test results of 0.79, and the Bartlett’s test of sphericity results of χ^2^(276) = 1313.24, p < 0.001, indicating that the data are adequate for factor analysis. Based on the eigenvalues and the scree plot, three possible models were found: with 3, 4, or 5 factors. These three models were explored with EFA which were conducted using a minimum residual estimation method and applying an Oblimin rotation. See S1 Table for the initial factor loading of the first EFA. To assure a good quality of the SOS scale, we established two criteria for the selection of items. First, items with low loading values (below 0.40) were progressively removed from the scale. Second, items with loading values above 0.40 in more than one component of the matrix, and therefore not specific to a single factor, were progressively deleted [48–51]. We first explored the 4 factors solution, which was in line with our hypothesized subscales. With this solution, the first factor was specific for the romantic partner body odor, the second for the familiar body odor, the fourth for the stranger body odor, whereas the third factor retained items that were not specific for the own body odor. For that reason, we also explored the 3 and 5 factors solutions. After Oblimin rotation, the structure that best fit the data was the 3 factors solution: KMO test: MSA = 0.78; Bartlett’s test: χ^2^(78) = 631.34, p < 0.001; Chi-squared test: χ^2^(42) = 71.84, p = 0.003. In total, 11 items were deleted and 13 were preserved (Table 1). Factor 1 contained 5 items and was loaded by romantic partner social odor items (called “PAR”), Factor 2 contained 4 items and was loaded by familiar social odor items (called “FAM”), Factor 3 contained 4 items and was loaded by stranger social odor items (called “STR”).

**Table 1:** Oblimin-rotated factor matrix (minimum residual method) of the 13 items.

Item n	Factor 1: PAR	Factor 2: FAM	Factor 3: STR	Uniqueness
**7**		0.683		0.480
**10**		0.588		0.545
**11**		0.662		0.514
**12**		0.790		0.424
**13**	0.514			0.510
**15**	0.592			0.674
**16**	0.840			0.316
**18**			0.445	0.740
**19**			0.678	0.539
**20**			0.543	0.708
**23**			0.470	0.761
**24**	0.718			0.393
**14**	-0.436			0.817

**Fig 1:**
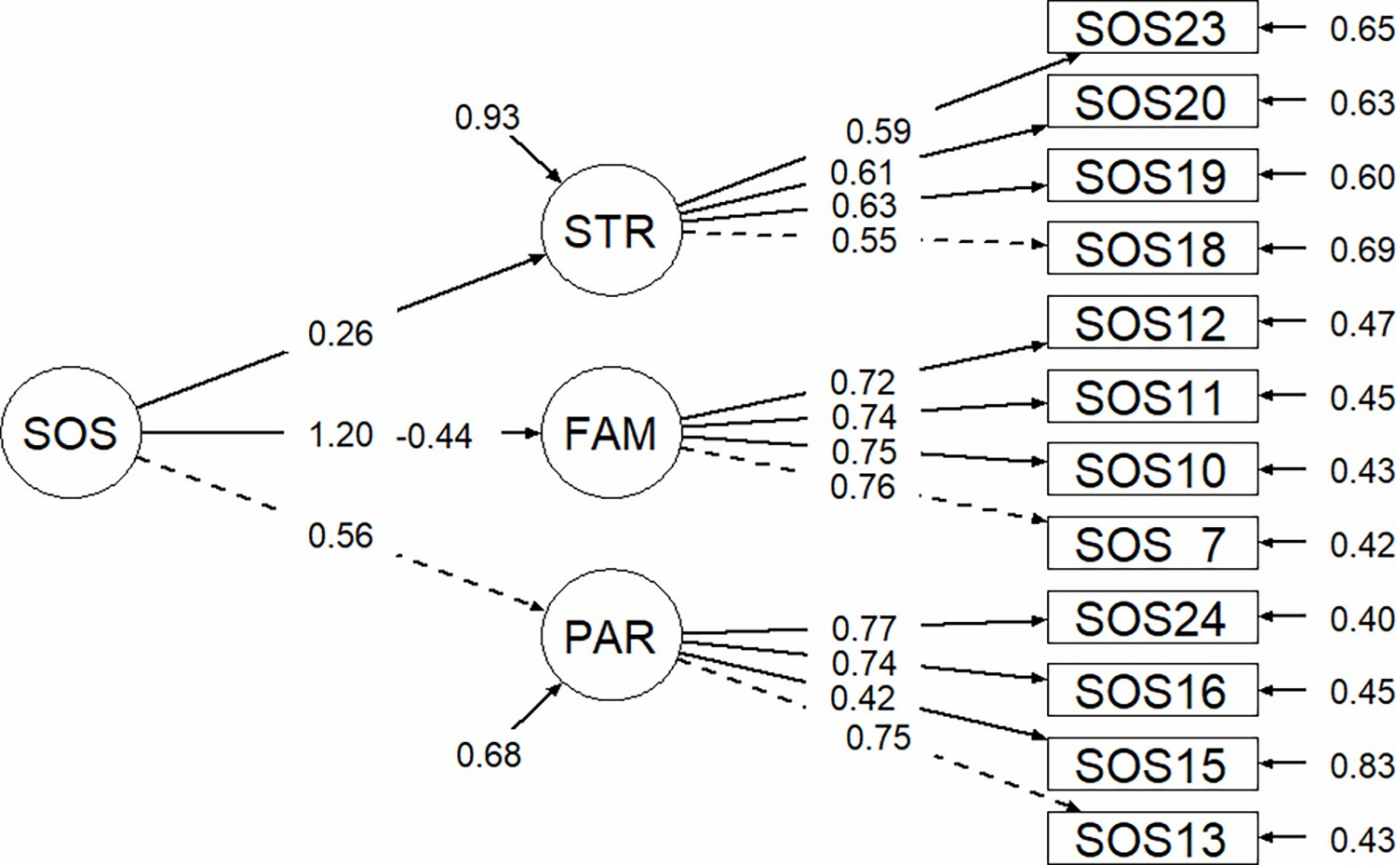
Factorial map of the CFA results for the SOS. CFA, confirmatory factor analysis; FAM, familiar social odor; PAR, romantic partner social odor; STR, stranger social odor.

The second-order confirmatory factor analysis (CFA) was performed to test the validity of the proposed 3-factor model and to examine if the three factors fitted the general concept of “social odor awareness”. As the items presented a non-normal distribution assessed with the Shapiro-Wilk test (all ps < 0.05), the diagonally weighted least squares (DWLS) was used for the CFA [52, 53]. The CFA provided the following goodness of fit indices: RMSEA (root mean square error of approximation) value was 0.038 (90% CI [0.000, 0.063]), SRMR (standardized root mean square residual) value was 0.077, CFI (Confirmatory Factor Index) value was 0.979, TLI (Tucker Lewis Index) value was 0.974. Although the model yielded a good fit to the data, the Factor 1 presented a poor reliability (McDonald’s ω coefficient = 0.66). Analysing the percentage of variance of each item that was explained by the factor, assessed by the squared standardized loadings of items, it was revealed that the item 14 poorly explained the Factor PAR (R^2^ = 0.105). For that reason, item 14 (see S1 Table) was removed from the scale and the CFA was conducted again. The final version of the scale presented a good fit (RMSEA = 0.012, SRMR = 0.069, CFI = 0.998, TLI = 0.997), as shown in Fig 1. Moreover, all the factors presented a good reliability (Factor PAR: McDonald’s ω = 0.77, Factor FAM: McDonald’s ω = 0.84, Factor STR: McDonald’s ω = 0.70; Total: McDonald’s ω = 0.80). See Table 2 for the inter-factor correlations of each item. The Italian translation of the SOS is available in S2 Table.

**Table 2:** Inter-factor correlation and median of the final version of the Italian scale.

Item n°	FAM	STR	PAR	Total
13			0.46	0.59
15			0.42	0.24
16			0.70	0.48
24			0.67	0.53
7	0.62			0.60
10	0.65			0.59
11	0.73			0.58
12	0.68			0.57
18		0.38		0.28
19		0.48		0.27
20		0.40		0.29
23		0.38		0.33
Median	0.67	0.39	0.56	0.52

## Study 2: Confirmatory factor analysis of the German version

Study 2 was designed to investigate the factor structure of the German version of the scale through the confirmatory factor analysis (CFA) and to clarify if it matches the previously found factor structure.

### Materials and methods

#### Participants

The Italian version of the SOS questionnaire was translated in German by an official Italian–German translator (the German translation of the SOS is available in S2 Table). Then, two native German speakers revised and approved the translation. The survey was presented online using the Qualtrics survey platform. Participants were recruited via social media, word of mouth and during classes at the University of Graz, University of Klagenfurt, and the Medical University of Vienna. A total of 359 German-speaking participants took part in this study. Inclusion and exclusion criteria were the same as Study 1. The final sample was composed of 211 participants (169 women, 36 men, 6 did not specify their gender), between the ages of 18–45 years (mean = 23.4; SD = 5.5).

#### Statistical analyses

CFA was performed similar to Study 1 aiming to evaluate whether the three-factor structure with 12 items found for the Italian version of the questionnaire can be replicated in the German version.

### Results and discussion

The CFA provided the following goodness of fit indices: RMSEA value was 0.024 (90% CI [0.000, 0.052]), SRMR value was 0.065, CFI value was 0.988, the TLI value was 0.984. Although the model yielded a good fit to the data, the reliability of the three factors is slightly lower than the original Italian version: Factor PAR, McDonald’s ω = 0.73, Factor FAM, McDonald’s ω = 0.71Factor STR, McDonald’s ω = 0.61, Total, McDonald’s ω = 0.70. See Table 3 for the inter-factor correlations of each item. See S3 Table for the final version of the German SOS scale and S4 Table for the English translation of the scale, which has not been validated.

**Table 3:** Inter-factor correlation of the final version of the German scale.

Item n°	FAM	STR	PAR	Total
13			0.41	0.49
15			0.38	0.24
16			0.62	0.49
24			0.59	0.48
7	0.47			0.37
10	0.29			0.33
11	0.56			0.36
12	0.56			0.34
18		0.42		0.35
19		0.36		0.21
20		0.33		0.19
23		0.45		0.25
Median	0.51	0.39	0.50	0.35

## Study 3: Association with the actual olfactory abilities

Study 3 was designed and pre-registered as part of a broader project that aims to explore if the awareness of body odors is related to the olfactory ability, awareness, and imagery, as well as to several psychological characteristics (https://osf.io/htdmw). We reported here the data related to the first part of the project (Hypothesis a) which had the aim of investigating whether social odor awareness is predicted by olfactory abilities as measured by Sniffin’ Sticks test [41].

### Participants, materials and procedure

Participants that were involved in previous projects that included the Sniffin’ Sticks test [41] at the University of Padova and at the University of Graz were contacted through emails and were asked to perform the SOS online (hosted by Qualtrics XM Platform). We initially contacted and invited 58 Italian and 104 Austrian participants between 18 and 35 years old. Exclusion criteria were chronic rhinitis or other conditions that may affect their ability to perceive odors, smoking, pregnancy or breastfeeding, presence of any severe somatic or neurological conditions, use of psychotropic drugs (including antidepressants, antipsychotics, anxiolytics and mood stabilizers) and being under a psychological therapy at the moment of the recruitment, presence of severe psychotic symptoms (i.e., hallucinations and/or delusions), presence of suicidal thoughts, incapability to understand and to give an informed consent for the experiment. Due to recent reports [42], all participants who were diagnosed with COVID-19 within the last 3 months before participating were excluded. A total of 57 participants (43 Italian and 13 Austrian) agreed to take part in this study. They were 43 women and 14 men, between the ages of 18–44 years (mean = 23.9; SD = 4.1).

#### Sniffin’ Sticks test

The computer-testing version of the Sniffin’ Sticks test was performed (Burghart Instruments, Wedel, Germany; [41]). The test is composed of three subtests assessing three different olfactory functions: 1) the odor detection threshold was determined for n-butanol with 16 stepwise dilutions, using the single staircase technique based on a three-alternative forced choice task (3AFC); 2) the odor discrimination was assessed over 16 trials again using a 3AFC task: for each discrimination step, three pens were presented in random order, two containing the same odor and the third containing the target odor; 3) the odor identification was measured by presenting 16 common odors, each presented with four verbal descriptors in a multiple forced-choice format (three distractors and one target). A total score (TDI) above 30.5 is considered to be within the normosmic range, whereas under 16.5 indicates the presence of anosmia [41]. According to these criteria, two anosmic participants were excluded. In order to obtain a wide variability of olfactory abilities related to social odor awareness, hyposmic participants were not excluded. The final sample was composed of 51 normosmic and 6 hyposmic.

### Statistical analyses and results

Statistical analyses were pre-registered at the Open Science Framework (OSF, (https://osf.io/htdmw, Hypothesis a). Data was cleaned and analyzed using the software R [45]. As control, a t-test (*t*.*test* function, *stats* package) was performed in order to assure that there were no differences between the two groups (Italian and Austrian) in terms of SOS total score. No significant differences were found between the Italian and the Austrian samples on the total score of the SOS [t(55) = 0.67, p = 0.50], for this reason we merged the two samples for all following analyses.

For the SOS total score a multiple regression model with TDI score as fixed factor was performed using the *lmer* function (*lme4* package). Gender, sexual orientation, and age were fitted as random intercepts. A second multiple regression model including discrimination, identification, and threshold scores was performed on the SOS total score. In order to dealing with singular fit, one random intercept and one TDI subscores at a time was removed from the model, and the resulting model was compared with the initial one on the basis of the AIC criterion [54], using the *anova* function (*lmerTest* package, [55]). In the final models, all random intercepts were removed, so linear regressions using the *lm* function were performed. For both models, no significant predictors were found (all F < 0.40, all p > 0.52). Additionally, the Bayesian equivalent of the two above regression models were performed using JASP [46]. For both models, results showed Bayes factors BF_10_ between 0.31 and 0.27 indicating anecdotal evidence for H_0_ [56]. These results suggest that SOS scores are not related to olfactory abilities measured with the Sniffin’ Sticks test, however further investigations are needed.

## Study 4: Relationship between SOS scale and demographic and olfactory measures

Study 4 was designed to clarify whether SOS scores are associated with demographic variables and other olfactory scales.

### Material and methods

#### Participants

Participants of Study 4 were taken from Studies 1, 2, and 3 who next to the SOS questionnaire completed additional scales. This dataset consisted of 286 participants from Study 1, 172 from Study 2, and 57 from Study 3. All these participants performed correctly all the 4 control items that were included among the proposed scales. Specifically, for Study 4, 12 participants were removed because they did not specify the gender. The final sample was composed of 503 participants (321 Italian, 182 Austrian; 396 women). Besides the SOS scale, participants were asked to complete the OAS, the AIO, the VOIQ and to answer some questions regarding socio-demographic information: age, gender, sexual orientation (collected on a 7-points Likert scale ranging from 0 = “Exclusively heterosexual” to 6 = “Exclusively homosexual”), smoking habits (“Do you smoke?”, “If yes, how many cigarettes per day?”), for women only, questions regarding the use of hormonal contraception and menstrual cycle. Demographic information can be found in Table 4.

**Table 4:** Demographic characteristics and questionnaires scores by the language of the participants.

N = 503	Italian participants (N = 321)	Austrian participants (N = 182)	t and p values
Female gender %	78	80	χ^2^ = 0.05, p = 0.82
Age	24.41 (4.84)	23.93 (5.94)	t = -0.92, p = 0.36
SOS	29.43 (7.05)	29.90 (6.16)	t = 0.75, p = 0.45
SOS Familiar	12.30 (3.45)	12.80 (2.88)	t = 1.75, p = 0.08
SOS Partner	9.83 (3.29)	9.81 (3.20)	t = -0.07, 0.94
SOS Strangers	7.29 (3.08)	7.28 (2.87)	t = -0.05, p = 0.96
OAS	115.33 (15.99)	115.56 (16.30)	t = 0.15, p = 0.88
AIO	2.21 (0.49)	2.23 (0.48)	t = 0.61, p = 0.54
VOIQ	57.51 (13.49)	56.09 (13.02)	t = -1.15, p = 0.25

*Notes*: Data are mean (Standard Deviation) of continuous and percentage (%) of categorical variables.

#### Self-reported olfactory measures (OAS, AIO, VOIQ)

To assess the relation between the SOS and the more general olfactory skills to environmental odors, participants were administered direct measures of self-reported olfactory function. Specifically, the Olfactory Awareness Scale (OAS, [2]) a 34-item questionnaire that evaluates the person’s tendency to pay attention to odorants in the environment. The Affective Importance of Odor scale (AIO, [57]) an 8-item questionnaire developed to study the role of odors in every-day life and, in particular, the impact of good and bad odors in affecting liking and memory for places, things and persons. The Vividness of Olfactory Imagery Questionnaire (VOIQ) measures the olfactory representation ability [58] presenting 16 scenes with different odors; here participants evaluate the vividness of the imagined odor using a 5-point scale (“1—perfectly realistic and as vivid as the actual odor” to “5—No odor at all, you only know that you are thinking of the odor”).

#### Statistical analyses

Data were cleaned and analysed using the software R [45]. Partial Pearson’s correlations (*rcorr* function, *Hmisc* package) and t-test (*t*.*test* function, *stats* package) were performed to investigate the association between demographic variables and the SOS scores. A p value of .05 was the cut-off for significance. The Benjamini–Hochberg procedure was applied to control the false discovery rate (FDR) where needed [59].

### Results

#### Demographic variables

The correlation analysis between the SOS total score and age did not yield a significant relationship (SOS total: r = 0.04; STR: r = 0.12; PAR: r = 0.09; FAM: r = -0.07). There was also a significant difference between women and men in the SOS total score [t(501) = 3.04, p (uncorr) = 0.002, p (FDR corr) = 0.005, d = 0.33] and in the FAM subscale [t(501) = 5.11, p (uncorr) < 0.001, p (FDR corr) < 0.001, d = 0.56]. No significant differences between gender and the other three subscales (ps > 0.05) were found. There were no significant correlations between sexual orientation and the SOS total score (ps > 0.05). Moreover, we investigated whether smoking habits were associated with lower scores of the SOS scale. For each participant, we gave 0 score if they reported not to be smokers, in case of smoking habits we reported the number of cigarettes smoked per day. However, no significant correlation was found between smoking habits and SOS scale (r = 0.07, p = 0.13).

#### Effects of reproductive state

Since previous studies have shown that olfactory perception, in particular for social odors [60], is affected by menstrual cycle phase [61, 62] and by the use of hormonal contraception [63], we analyzed the differences in the SOS scores related to these variables. There were no significant differences in the SOS score between women using hormonal contraception and those not using it [t(383) = 0.79, p = 0.43)]. To investigate the effect of the menstrual phase, the cycle length of each woman not taking hormonal contraception was standardized to a 28-day cycle by adjusting the follicular phase of each woman with respect to the length of her normal menstrual cycle [64]. The group was then classified into the follicular (starts on the first day of menstruation and ends with ovulation; < 15 days; n = 142) or luteal (latter phase of the menstrual cycle; ≥ 15 days; n = 143) [65, 66].

**Fig 2:**
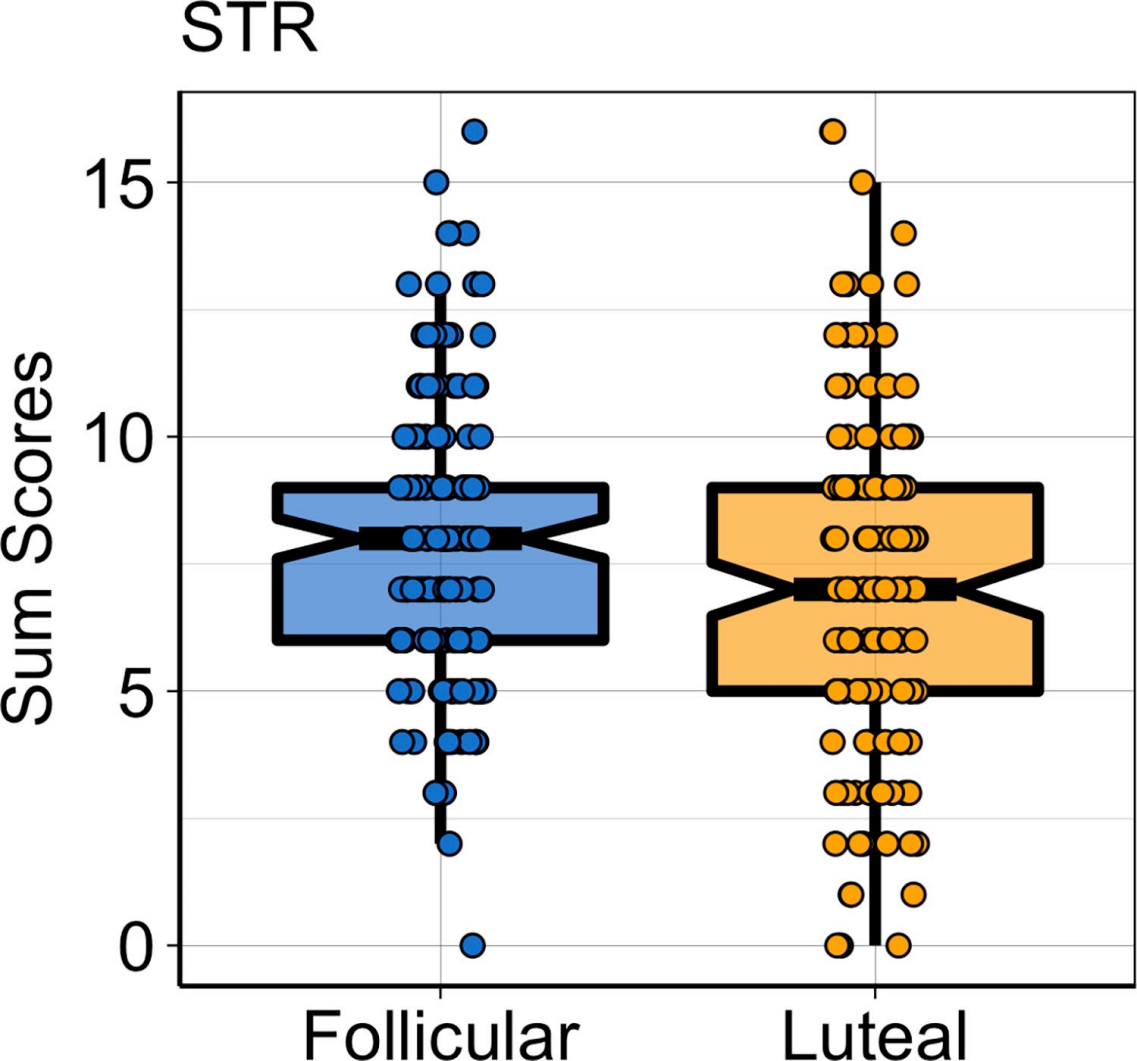
Data distribution for stranger social odor subscale. Boxplots depict the median (horizontal black line) and quartile ranges of the distribution, whiskers indicate maximum and minimum values, coloured dots represent data distribution.

Even though the difference of the SOS total score was not significant [t(283) = 1.80; p = 0.07, p-FDR = 0.10, d = 0.21], there was a trend toward significance for the STR subscale [t(283) = 2.27; p (uncorr) = 0.02, p (FDR corr) = 0.06, d = 0.27; Fig 2], with women in the follicular phase reporting higher awareness for stranger odors (M = 7.79, SD = 2.79) than women in the luteal phase (M = 6.97, SD = 3.31). No significant differences were found for FAM and PAR subscales (ps FDR corr > 0.05).

#### Olfaction measures

**Fig 3:**
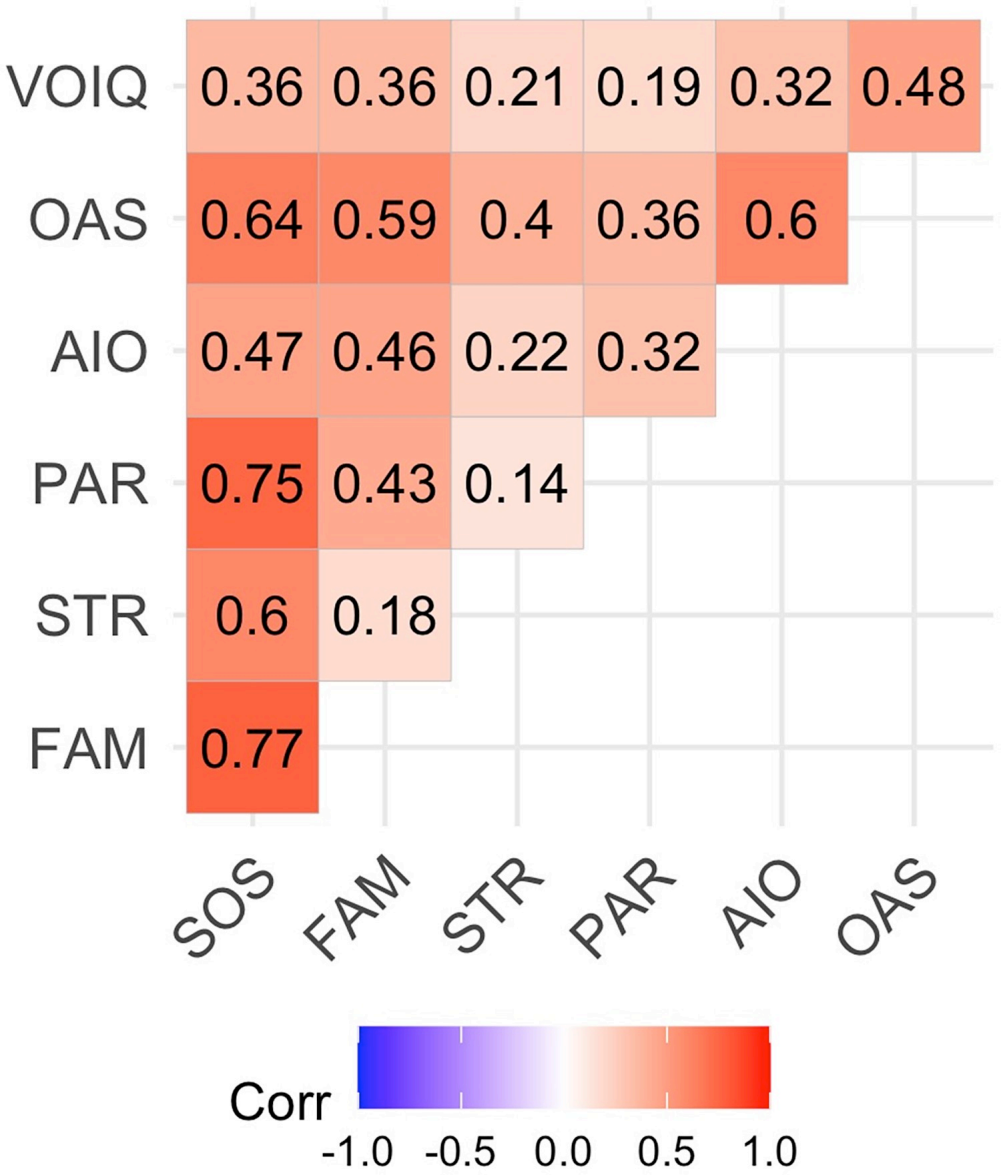
Graphical display of the correlation matrix between OAS, AIO, and VOIQ scores and the SOS score and its subscales. Positive correlations are displayed in red and negative correlations in blue colour. Colour intensity is proportional to the correlation coefficients. Correlation coefficients are displayed inside the boxes.

The correlation analyses between the SOS and its three subscales and the olfactory measures (OAS, AIO, and VOIQ) confirmed that the scale has a good correlation with measures of awareness of olfactory stimuli and the affective response to them. Indeed, the total score of the SOS was significantly positively correlated, with medium/large effect sizes, with the OAS (r = 0.64, p < 0.001), with the AIO (r = 0.47, p < 0.001), and the VOIQ (r = 0.36, p < 0.001). Some subscales of SOS were also significantly positively correlated, with medium/large effect sizes, with the OAS (FAM: r = 0.59, p < 0.001; PAR: r = 0.36, p < 0.001; STR: r = 0.40, p < 0.001), with the AIO (FAM: r = 0.46, p < 0.001; PAR: r = 0.32, p < 0.001), and with VOIQ (FAM: r = 0.36, p < 0.001). Other subscales were correlated, but with small effect sizes, with the AIO (STR: r = 0.22, p < 0.001), and with VOIQ (PAR: r = 0.19, p < 0.001; STR: r = 0.21, p < 0.001). See Fig 3.

## General discussion

Despite the growing body of literature documenting the importance of human social odors on everyday social interactions [4] as well as the presence of alteration of social odor perception in neuropsychiatry diseases [67, 68] and neurodevelopmental disorders [69, 70], the attention humans pay to social odors has been largely neglected in the available measurements of odor awareness. Having a proper scale developed to assess social odor awareness is thus extremely relevant for the understanding of social odor processing and the social related behavior.

Here, we have developed and validated across 4 studies a new questionnaire called Social Odor Scale (SOS) designed to specifically assess social odor awareness. Initially, we hypothesized a four-factor structure of the SOS, as the scale was developed to measure the level of attention participants pay to the four types of social odors (own, familiar, romantic partner, and stranger) that occur in individuals’ everyday life. From the exploratory factor analysis, the factors extracted did not completely match our initial hypothesis, indeed there was no statistical support for the subscale regarding the own body odor. For that reason, we adjusted the model according to a 3-factor structure: Familiar Social Odors, Romantic Partner Social Odor, Stranger Social Odor. The CFA confirmed the validity of the second-order model with one higher-order factor and three lower-order factors. The higher order factor represents social odor awareness as a whole, whereas the obtained three lower-order factors represent the awareness of Familiar Social Odors, Romantic Partner Social Odor, and Stranger Social Odor. The results of Studies 1 and 2 indicated that this new scale had a good validity and an overall good reliability to measure social odor awareness in Italian and Austrian samples.

Contrary with our hypothesis, in Study 3 it was found that the olfactory abilities did not predict the score on the SOS questionnaire. Results on the relation between odor awareness and olfactory performance are mixed. Indeed, even though some studies reported a positive relation between odor awareness and olfactory performance [2, 71], when age is controlled, this positive association disappeared [71]. Similarly, both in adults and in children, no association has been found between odor identification performance and self-reported odor awareness [72, 73]. Our result is in line with the notion that people with undetected anosmia or hyposmia do not consciously perceive their impairment and, therefore, are able to correctly read and recognize social cues, maybe with the integration from other sensory modalities [74]. Hence, it seems that the olfactory metacognitive abilities associated with social odors are not linked to the actual olfactory performance.

In Study 4, the final aim was to clarify whether SOS scores are associated with demographic variables and other widely used olfactory scales (i.e., OAS, AIO, and VOIQ). Results showed that women paid more attention to social odors and, in particular, to familiar body odors than men. This result is in line with studies showing a gender difference in various metacognitive abilities related to odors. For instance, women reported higher odor awareness [72], higher interest in the sense of smell [75], and higher importance of olfaction [21, 29] than male. This is in addition to an overall better olfactory performance [76]. Among women, there was no effect of oral contraception use in social odor awareness measured with the SOS. Moreover, albeit there is only a trend toward significance, women in the follicular phase reported a numerically higher awareness for stranger social odors (i.e., higher SOS stranger subscale) than women in the luteal phase. Accordingly, previous works reported higher sensitivity toward body odors during the follicular phase, compared with the luteal one [77, 78]. Our results highlight the need of further studies to better clarify the role of the reproductive menstrual phase in social odors’ processing.

Finally, the SOS total score and its subscales were found to be closely related to all the olfactory measures taken into account. This result further validates the SOS and confirms its role as a new measure of odor awareness. Indeed, human’s ability to pay attention to social odor is a particular component of the general awareness toward olfactory contents that, as presented by our results, is modulated by specific individual characteristics (i.e., age, gender and reproductive state). However, further studies are warranted to investigate how the SOS scale relates to the actual perception of social odors (in particular stranger, familiar and partner social odors) to provide stronger evidence in favour of the scale’s validity.

In the present work some limitations have to be acknowledged. First, the SOS questionnaire has been performed online, limiting the experimental control toward the respondents. However, we included in the survey control items to assess that participants properly read the items’ content. Moreover, SOS scores reflect self-perceived behavior rather than behavior itself and should be affected by response bias, an issue relative to all assessments involving self-reports. Secondly, participants were relatively young and most of them were female, making it impossible to generalize the current results to the general population. However, as it is widely reported that olfactory abilities decline with age [79, 80], we included only participants with an age between 18 and 45 years to have a homogeneous sample and to minimize the impact of age on the scoring on the SOS.

Future works should relate the SOS to the actual abilities to perceive body odors, as well as to the behavioral and physiological responses to them. This multimodal assessment could be helpful to achieve a better understanding of the relation between self-reported responses, cognitive processes, physiological and behavioral measurements of body odor-related responses. Moreover, SOS should also be examined in clinical populations characterized by social impairments, such as depression and social anxiety, increasing our understanding of these complex disorders. Certainly, addressing these issues may help further clarify the role of social odors in humans.

In conclusion, the SOS was developed to fill the gap left by the existing scales that neglected the multifaceted functions of body odors in social interactions. The SOS is useful to assess the social awareness toward three types of social odor (familiar, romantic partner and stranger body odor) that are relevant in our everyday life. Odors and social odors were found to be altered in several neurological and psychiatric diseases including Alzheimer [81], Parkinson [82] and Autism [69, 70], affective disorders [67, 68, 83] and schizophrenia [84], the use of this questionnaire will provide a useful assessment tool which may help to disentangle the role of such alterations in neuropsychiatric conditions.

## Supporting information

S1 Table Initial factor loading of the 24 items version in Study 1. (PDF)

S2 Table Final Italian version of the SOS. (PDF)

S3 Table Final German version of the SOS. (PDF)

S4 Table Final English version of the SOS. This version has not been validated.
(PDF)
