# Supplementary material for: The social odor scale: Development and initial validation of a new scale for the assessment of social odor awareness
Source: PLoS One. 2021 Dec 14;16(12):e0260587. doi: 10.1371/journal.pone.0260587 (PMC8670672; doi:10.1371/journal.pone.0260587)
Supplement: S1 Table — (PDF) [file pone.0260587.s001.pdf]

S1 Table. Initial factor loading of the 24 items version in Study 1.

Item n°	Factor 1	Factor 2	Factor 3	Factor 4	Uniqueness
1. I smell my clothes to see if I should wash them or not	0.385	0.077	-0.156	0.196	0.787
2. I smell my clothes before I put them on	0.385	0.239	-0.032	0.225	0.662
3. I think about how other people perceive my body odor	0.180	0.116	0.057	0.470	0.685
4. I like my body odor without deodorant or perfume	0.246	0.161	-0.026	-0.186	0.850
5. I think about how members of the opposite sex perceive my odor	0.199	0.022	-0.073	0.656	0.548
6. I have a tendency of bringing often my hands close to my face when I'm in an unfamiliar situation	0.122	0.225	-0.194	-0.030	0.899
7. I can recognize people by their odor	0.022	0.690	0.083	-0.048	0.479
8. I prefer that other people hide their body odor with deodorant or perfume	-0.188	-0.219	0.284	0.388	0.663
9. The odor of a person is important to understand whether I like that person or not	0.267	0.328	0.209	0.227	0.564
10. I can relax when I smell someone I care about	0.150	0.591	-0.042	-0.049	0.566
11. I have well imprinted in my mind the odor of certain people	0.020	0.616	0.119	0.048	0.542
12. Smells can make me remember people I haven't seen in a long time	-0.068	0.770	-0.060	-0.000	0.470
13. I can be attracted to someone for their body odor	0.583	0.229	-0.005	0.235	0.428
14. I prefer my sexual partner to hide their body odor with deodorant or perfume	-0.336	-0.080	0.051	0.616	0.469
15. I like the way my partner's armpits smell	0.557	-0.025	0.009	-0.320	0.589
16. I can be sexually aroused by someone's natural body odor	0.790	-0.111	0.082	-0.003	0.427
17. My partner's smell or scent can inhibit my sexual behavior	-0.086	0.084	0.127	0.387	0.792
18. In a public place (e.g. the movie theater), I look for another place to sit if a person has an unpleasant odor	0.145	0.062	0.499	0.116	0.649
19. I don't take public transport because of the odor of other people	0.205	-0.109	0.443	0.045	0.770
20. When I enter in a crowded room, I ask if	0.110	-0.088	0.437	0.073	0.793

it is possible to open the windows					
21. The smell or scent of other people can be repulsive or sickening	0.020	0.270	0.344	0.049	0.739
22. When I’m close to strangers I notice if their shirt has a strong smell	-0.037	0.420	0.482	0.035	0.484
23. I get quickly annoyed by the odor of strangers	-0.025	0.018	0.700	-0.098	0.527
24. I can be aroused by my sexual partner’s natural body odor	0.687	0.150	0.012	-0.063	0.408
