# Supplementary material for: The social odor scale: Development and initial validation of a new scale for the assessment of social odor awareness
Source: PLoS One. 2021 Dec 14;16(12):e0260587. doi: 10.1371/journal.pone.0260587 (PMC8670672; doi:10.1371/journal.pone.0260587)
Supplement: S2 Table — (PDF) [file pone.0260587.s002.pdf]

S2 Table. Final Italian version of the SOS.

Indichi quanto è d’accordo o disaccordo con ciascuna delle seguenti affermazioni, segnando una casella corrispondente. Scelga l’opzione centrale soltanto se davvero non è nella possibilità di valutare il suo comportamento.	Non sono per niente d'accordo	Non sono molto d'accordo	Non sono né d'accordo e né in disaccordo	Sono abbastanza d'accordo	Sono completamente d'accordo
1. Riesco a riconoscere le persone dal loro odore					
2. Riesco a rilassarmi quando sento l'odore di qualcuno per cui provo affetto					
3. Ho bene impresso nella mia mente l'odore di certe persone					
4. Gli odori possono suscitare in me il ricordo di persone che non vedo da tempo					
5. Posso essere attratto da qualcuno per il suo odore corporeo					
6. Mi piace il modo in cui le ascelle del mio partner odorano					
7. Posso essere eccitato sessualmente dall'odore naturale del corpo di qualcuno					
8. Posso essere attratto dall’odore naturale del mio partner sessuale					
9. In luogo pubblico (es. al cinema) se una persona ha un odore sgradevole cerco un altro posto a sedere					
10. Non prendo mezzi pubblici a causa dell'odore degli altri					
11. Quando entro in una stanza affollata, mi informo se è possibile arieggiare aprendo le finestre					
12. Sono infastidito velocemente dall'odore degli estranei					
