# Supplementary material for: The social odor scale: Development and initial validation of a new scale for the assessment of social odor awareness
Source: PLoS One. 2021 Dec 14;16(12):e0260587. doi: 10.1371/journal.pone.0260587 (PMC8670672; doi:10.1371/journal.pone.0260587)
Supplement: S3 Table — (PDF) [file pone.0260587.s003.pdf]

S3 Table. Final German version of the SOS.

Geben Sie an, in welchem Maße Ihre persönliche Art zu reagieren damit übereinstimmt, was in der Aussage steht. Die Mitte der Skala gebrauchen Sie nur, wenn Sie die beschriebene Reaktion überhaupt nicht beurteilen können.	Ich bin überhaupt nicht einverstanden	Ich bin nicht sehr einverstanden	Ich bin weder einverstanden noch dagegen	Ich bin ziemlich einverstanden	Ich bin völlig einverstanden
1. Ich kann Personen nach ihrem Geruch unterscheiden					
2. Ich kann mich entspannen beim Duft einer Person, für die ich positive Gefühle habe					
3. Der Geruch gewisser Personen bleibt mir gut in Erinnerung					
4. Gerüche können mich an Personen erinnern, die ich lange nicht gesehen habe					
5. Ich kann mich zu jemand hingezogen fühlen wegen seines Körpergeruchs					
6. Ich mag es, wie die Achseln meines Partners riechen					
7. Der natürliche Körpergeruch von jemand kann mich sexuell erregen					
8. Ich kann mich vom natürlichen Geruch meines Sexualpartners angezogen fühlen					
9. Wenn eine Person an einem öffentlichen Ort (z.B. im Kino) unangenehm riecht, suche ich mir einen anderen Sitzplatz					
10. Ich benütze keine öffentlichen Verkehrsmittel wegen des Geruchs der anderen					

11. Wenn ich in einen überfüllten Raum komme, frage ich, ob man die Fenster öffnen und lüften kann					
12. Der Geruch von Fremden geht mir schnell auf die Nerven					
