# Supplementary material for: The social odor scale: Development and initial validation of a new scale for the assessment of social odor awareness
Source: PLoS One. 2021 Dec 14;16(12):e0260587. doi: 10.1371/journal.pone.0260587 (PMC8670672; doi:10.1371/journal.pone.0260587)
Supplement: S4 Table — This version has not been validated. (PDF) [file pone.0260587.s004.pdf]

S4 Table. Final English version of the SOS. This version has not been validated.

Indicate how much you agree or disagree with each of the following statements by ticking a corresponding box. Choose the central option only if you are really unable to evaluate your behavior.	I totally disagree	I mostly disagree	I neither agree nor disagree	I mostly agree	I totally agree
1. I can recognize people by their odor					
2. I can relax when I smell someone I care about					
3. I have well imprinted in my mind the odor of some people					
4. Odors can evoke in me the memory of people I have not seen for a long time					
5. I can be attracted to someone for their body odor					
6. I like the way my partner's armpits smell					
7. I can be sexually aroused by someone's natural body odor					
8. I can be aroused by my sexual partner's natural body odor					
9. In a public place (e.g. the movie theater), I look for another place to sit if a person has an unpleasant odor					
10. I don't take public transport because of the odor of other people					
11. When I enter in a crowded room, I ask if it is possible to open the windows					
12. I get quickly annoyed by the odor of strangers					
